# Evaluation of Artificial Intelligence as a Decision-Support Tool in Urological Tumor Boards: A Study in Real Clinical Practice

**DOI:** 10.3390/jcm15062130

**Published:** 2026-03-11

**Authors:** Javier De la Torre-Trillo, Yaiza Yáñez Castillo, Maria Teresa Melgarejo Segura, Elisa Carmona Sánchez, Alberto Zambudio Munuera, Juan Mora-Delgado, Alfonso López Luque

**Affiliations:** 1 Urology Department, Hospital Regional Santa Ana, Av. Enrique Martín Cuevas, s/n, 18600 Motril, Spain; ymyc10@gmail.com (Y.Y.C.); lisscarsan@gmail.com (E.C.S.); allo2198@hotmail.com (A.L.L.); 2 Grupo URO-MET, Instituto IBS Granada, Avda. de Madrid, 15, 18012 Granada, Spain; mtm.segura@gmail.com; 3 Urology Department, Hospital Universitario San Cecilio, Av. de la Investigación, 18016 Granada, Spain; alberto.zambudiomunuera@gmail.com; 4 Infectious Diseases and Clinical Microbiology Department, Hospital Jerez de la Frontera, Ctra. Trebujena, s/n, Jerez de la Frontera, 11407 Cadiz, Spain; juanmorainternista@gmail.com

**Keywords:** artificial intelligence, urological cancer, tumor board, ChatGPT, clinical decision-making, machine learning

## Abstract

**Background/Objectives**: Artificial intelligence (AI) tools, particularly large language models (LLMs) such as ChatGPT-4o, are gaining prominence in medicine. While their diagnostic capabilities have been explored across various oncologic domains, their role in clinical decision-making within multidisciplinary tumor boards (MTBs) remains largely unexamined in urologic oncology. This study evaluates the performance of ChatGPT-4o as a decision-support tool in a real-world MTB setting by comparing its recommendations with those of expert clinicians. **Materials and Methods**: A retrospective study was conducted using 98 anonymized clinical cases discussed by a urologic MTB between June 2024 and February 2025. An independent urologist entered the same cases into ChatGPT-4o using a standardized prompt replicating real-world presentation. Two certified urologists independently assessed the model’s responses. Agreement was analyzed overall and by tumor type, disease stage, clinical context, and treatment strategy. **Results**: ChatGPT-4o fully agreed with the MTB in 56.1% of cases, was correct but incomplete in 23.5%, and provided partially accurate but flawed recommendations in 18.4%. Overall concordance between ChatGPT-4o and the MTB yielded a Cohen’s kappa of 0.61, indicating moderate-to-good agreement. Discrepancies were most common in metastatic prostate cancer, often due to misclassification of tumor burden or errors in treatment sequencing. Highest agreement rates were observed in bladder and renal tumors, and in standardized therapeutic scenarios such as radiotherapy. **Conclusions**: ChatGPT-4o demonstrated moderate alignment with expert MTB decisions and performed best in well-defined clinical contexts. While it cannot replace multidisciplinary expertise, it may serve as a supportive tool to enhance access to standardized oncologic care.

## 1. Introduction

Over the past decade, the integration of artificial intelligence (AI) into healthcare has accelerated considerably, with applications spanning diagnostic imaging, prognostic modeling, treatment planning, and workflow optimization [1,2]. Advances in machine learning, particularly deep learning, have enabled the development of systems capable of identifying complex patterns within large-scale datasets and assisting clinicians in data interpretation and decision-making processes [3]. As healthcare systems face increasing patient volumes and growing complexity of oncologic care, interest in AI-driven decision-support tools has intensified.

Among these technologies, large language models (LLMs), such as ChatGPT (developed by OpenAI), have attracted substantial attention. These models are trained using large-scale neural architectures on extensive textual corpora and are designed to generate coherent, context-aware responses across diverse domains [4]. Unlike traditional rule-based systems, LLMs rely on probabilistic language modeling to synthesize information and simulate structured reasoning. More recent iterations, including ChatGPT-4o, have demonstrated improvements in contextual understanding, reasoning consistency, multilingual processing, and response latency compared with earlier versions. These advancements have led to growing exploration of their potential roles in medical education, patient counseling, clinical documentation, and decision support [1].

However, despite promising capabilities, several limitations constrain their immediate clinical deployment. LLMs may produce factually incorrect or fabricated information, lack transparent reasoning pathways, and demonstrate variability in output depending on prompt formulation or model parameters. Furthermore, their performance may not consistently align with established clinical guidelines, particularly in complex scenarios. These concerns underscore the necessity of systematic, case-based validation studies before considering integration into real-world clinical workflows [5,6].

Clinical decision-making in urologic oncology is inherently multifaceted and often requires multidisciplinary collaboration. Multidisciplinary Tumor Boards (MTBs) represent a cornerstone of contemporary oncologic care and are widely regarded as a standard approach for complex case management. These meetings bring together urologists, medical oncologists, radiation oncologists, radiologists, pathologists, and pharmacists to integrate clinical findings, imaging results, histopathology, patient comorbidities, and guideline recommendations into individualized treatment strategies. Evidence suggests that MTBs can improve adherence to evidence-based guidelines, optimize sequencing of therapies, and enhance interprofessional communication. Nevertheless, MTBs are resource-intensive and logistically demanding. They require coordination across multiple specialties, dedicated scheduling time, and access to comprehensive patient data. Increasing case volumes, workforce limitations, and institutional variability may affect the consistency and depth of discussions. In some settings, limited availability of subspecialized expertise may further constrain optimal multidisciplinary deliberation. These operational challenges have prompted interest in tools capable of supporting or augmenting multidisciplinary decision-making processes [7,8].

In this context, AI may represent a valuable tool to help standardize decision-making, reduce clinician workload, and serve as a supportive system in complex clinical scenarios.

To date, several studies have assessed ChatGPT’s performance across various oncologic domains—such as breast, head and neck, sarcoma, gastrointestinal, gynecologic, and molecular oncology—with variable results [9,10,11,12,13,14].

Despite the growing body of literature examining AI in oncology, the application of LLMs within urologic oncology has not been systematically evaluated in direct comparison with authentic tumor board decisions derived from routine clinical practice. Urologic oncology presents unique challenges, including heterogeneous disease stages, evolving therapeutic landscapes such as targeted therapies and immunotherapies, and nuanced risk stratification systems. Assessing AI-generated recommendations against real multidisciplinary consensus decisions therefore represents a more stringent and clinically meaningful validation framework than comparisons based solely on guideline queries.

The present study aims to evaluate the concordance between recommendations generated by ChatGPT-4o and those issued by an expert multidisciplinary urologic oncology tumor board across a series of real clinical cases. By directly comparing AI-generated outputs with consensus-based clinical decisions, this study seeks to quantify agreement, identify domains of divergence, and explore the current capabilities and limitations of LLMs as potential decision-support tools in urologic oncology.

## 2. Materials and Methods

### 2.1. Study Design

A retrospective cross-sectional comparative study was conducted to evaluate the concordance between decisions made by a multidisciplinary urologic oncology tumor board in routine clinical practice and recommendations generated by ChatGPT-4o (OpenAI, San Francisco, CA, USA), using the same clinical cases discussed at a hospital-based MTB in southern Spain between June 2024 and February 2025. All data were irreversibly anonymized, and the study was approved by the local ethics committee (SICEIA-2025-001653).

### 2.2. Inclusion and Exclusion Criteria

Patients with a histopathological diagnosis of prostate, bladder, kidney, testicular, or upper urinary tract cancer were eligible. Patients with rare urologic tumors (defined as incidence below 2 percent), those previously treated at other centers, or those referred exclusively for subsequent treatment were excluded. In our institution, MTB discussions are primarily reserved for complex cases requiring multidisciplinary evaluation, including cases involving multimodal treatment strategies, diagnostic uncertainty, or advanced-stage disease. Straightforward cases that clearly follow guideline-based pathways are generally not presented at the MTB and were therefore not included in this analysis.

### 2.3. Variables

The following variables were extracted from the electronic medical records: age, sex, Charlson Comorbidity Index, tumor type and stage, reason for case presentation (diagnosis, treatment, or follow-up), and the MTB’s final decision.

### 2.4. Method and Outcomes

Clinical cases were discussed during regular MTB meetings, and MTB decisions were categorized as diagnostic, therapeutic, or follow-up. Subsequently, an independent urologist who did not participate in the MTB discussions entered the same cases into ChatGPT-4o using a previously validated prompt replicating the structure and level of detail typically presented during MTB meetings. The full structured prompt and a representative example of case input are provided as Supplementary Material.

To minimize potential learning or memory bias, each case was entered in separate sessions held on different days, without cumulative input or feedback across sessions. The study did not perform multiple repeated runs for identical inputs, as the objective was to simulate a single-instance clinical decision-support interaction.

Two board-certified urologists independently assessed ChatGPT-4o responses using a standardized four-point scale (Table 1). When discrepancies occurred, evaluators discussed the case and agreed on a consensus classification. Only the final consensus score was recorded; therefore, inter-rater agreement statistics could not be calculated because the individual ratings were not retained.

The primary outcome was the level of agreement between ChatGPT-4o and MTB decisions. Secondary outcomes included agreement stratified by tumor type, stage, clinical context (diagnostic, therapeutic, or follow-up), and treatment strategy (surgery, radiotherapy, hormonotherapy, immunotherapy).

The tumor board’s final decision was considered the reference standard (gold standard) against which ChatGPT-4o responses were compared. Neither evaluator was involved in the prompt design or in the original MTB discussions, ensuring an independent assessment. Specific details regarding medications, surgical techniques, or radiotherapy modalities were not included in the comparative analysis.

### 2.5. AI Statement

ChatGPT-4o was used as the object of evaluation in this study. The model was queried using a standardized prompt and anonymized clinical case summaries derived from routine multidisciplinary tumor board discussions. The present study did not involve model training, architectural modification, hyperparameter tuning, loss-function implementation, or optimization procedures. ChatGPT-4o was used as a proprietary large language model operating as a closed black-box system, and its internal architecture, training data, and parameter configuration are not accessible to end users and were not modified for the purposes of this study. The AI system was not involved in data analysis, statistical processing, or manuscript drafting beyond its role as the evaluated intervention. All clinical interpretations, methodological decisions, and final conclusions were independently performed and validated by the authors.

### 2.6. Statistical Analysis

Descriptive statistics were used to summarize patient and tumor characteristics. Continuous variables are presented as mean and standard deviation, while categorical variables are reported as absolute frequencies and percentages.

Agreement between ChatGPT-4o recommendations and multidisciplinary tumor board decisions was assessed using Cohen’s kappa coefficient to evaluate concordance beyond chance. The strength of agreement was interpreted according to conventional thresholds: values below 0.20 were considered poor, 0.21 to 0.40 fair, 0.41 to 0.60 moderate, 0.61 to 0.80 good, and greater than 0.81 very good agreement.

All statistical analyses were performed using Python 3.13. A two-sided *p*-value < 0.05 was considered statistically significant.

## 3. Results

### 3.1. Population Characteristics

A total of 130 clinical cases were analyzed, of which 98 met the inclusion criteria. The mean patient age was 69.98 years, and the majority were male (95.9%). The mean Charlson Comorbidity Index was 4.38. Regarding tumor distribution, 67.3% of cases involved prostate cancer, followed by bladder (22.4%), renal (8.2%), and testicular or upper urinary tract tumors (1% each). Overall, 58.2% of tumors were localized, while 41.8% were metastatic.

### 3.2. Overall Agreement Between ChatGPT-4o and the Tumor Board

**Figure 1:**
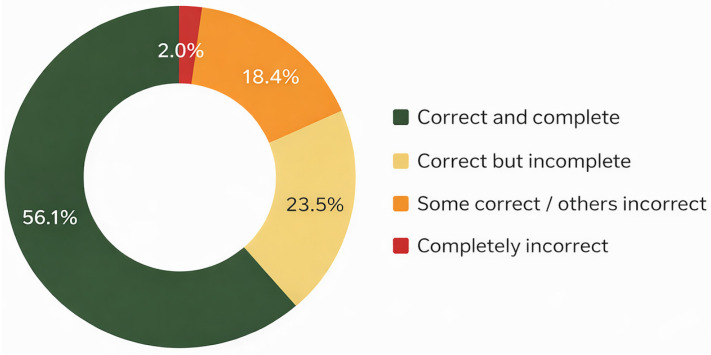
Agreement Levels Between ChatGPT-4o Recommendations and Tumor Board Decisions.

Of the 98 evaluated cases, ChatGPT-4o fully matched the tumor board’s decision in 55 cases (56.1%; 95% CI: 46.3–65.5%). In 23 cases (23.5%; 95% CI: 16.2–32.8%), responses were correct but incomplete, and in 18 cases (18.4%; 95% CI: 11.9–27.2%), the model included accurate elements along with erroneous recommendations (Figure 1). A categorical comparison between the MTB final decisions and ChatGPT-4o recommendations yielded a Cohen’s kappa of 0.61 (95% CI: 0.51–0.71), indicating moderate-to-good agreement according to standard interpretation thresholds. Only two cases (2.0%; 95% CI: 0.6–7.1%) were completely discordant. Both corresponded to complex prostate cancer scenarios: a patient with castration-resistant prostate cancer in fourth-line therapy, for whom the model proposed a new antiandrogen despite being in a context of therapeutic limitation; and a case of biochemical recurrence after radiotherapy in which ChatGPT-4o failed to include salvage prostatectomy as a treatment option.

### 3.3. Agreement by Tumor Type, Stage, and Clinical Context (Figure 2)

Agreement between ChatGPT-4o and the MTB showed descriptive variations according to tumor type, disease stage, and clinical context. However, statistical analysis using the Chi-square test (X^2^ = 10.08; *p* = 0.6088) and Fisher’s exact test (*p* = 0.4657) did not reveal significant differences between the level of agreement and these variables.

In prostate cancer ChatGPT-4o showed complete agreement in 53% of cases, incomplete agreement in 24.2%, partially correct responses in 19.7%, and total discordance in 3%. For localized disease, full agreement reached 60.5%, while in metastatic disease it dropped to 42.9%. In several of these cases, errors were related to misclassification of metastatic volume according to the CHAARTED criteria [15], which impacted systemic therapy selection. In bladder tumors, overall agreement was 63.6%, reaching 66.7% in metastatic cases. In renal tumors, total agreement was 62.5%. A single testicular tumor case achieved complete agreement, and one upper urinary tract tumor showed partial concordance.

Regarding the reason for case presentation, higher agreement was observed in therapeutic decisions, particularly for bladder (66.7%) and prostate (62.1%) tumors. In contrast, diagnostic and follow-up discussions showed lower agreement rates, generally below 50%, except for one bladder cancer case, which showed complete agreement (100%).

**Figure 2:**
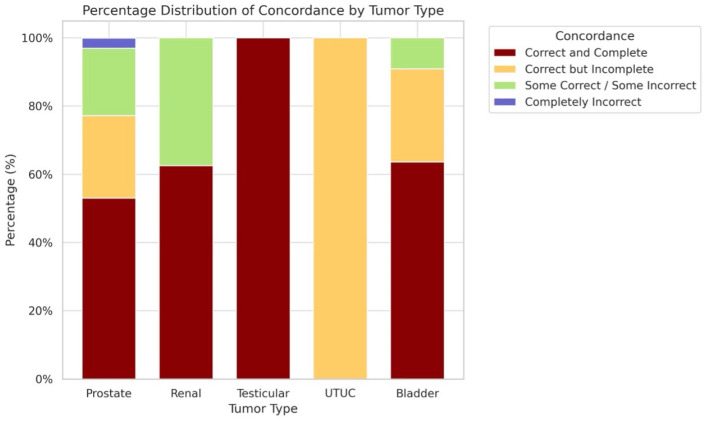
Agreement between ChatGPT-4o recommendations and tumor board decisions by tumor type.

### 3.4. Agreement by Treatment Strategy

**Figure 3:**
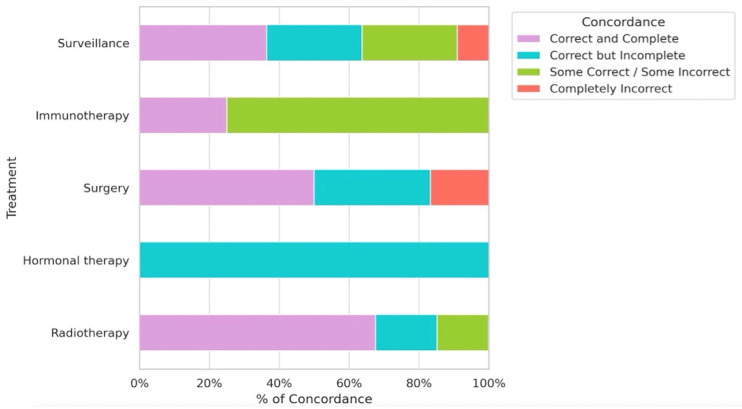
Agreement Between ChatGPT-4o Recommendations and Tumor Board Decisions by Treatment Strategy.

When analyzed by treatment modality (Figure 3), the highest agreement was observed in cases involving radiotherapy: 67.6% full agreement, 17.6% correct but incomplete responses, and 14.7% partially correct answers, with no fully incorrect recommendations.

In hormonotherapy cases, all responses were correct but incomplete, indicating that the model captured the general therapeutic intent but lacked specificity regarding treatment details. In surgical cases, full agreement was observed in 50% of cases, with 33.3% showing partially correct responses and 16.7% including erroneous recommendations. Performance was lower in immunotherapy cases, with only 25% full agreement and 75% of responses containing inaccurate elements. In active surveillance scenarios, the model demonstrated intermediate performance: 36.4% full agreement, 27.3% partial agreement, 27.3% incorrect responses, and 9.1% complete discordance. For complementary diagnostic tests such as PET-PSMA or PET-choline, agreement rates were below 50%, especially in cases involving PET-choline indications.

## 4. Discussion

The use of AI in urology, particularly in oncology, is rapidly expanding. Most studies to date have focused on diagnostic applications. One notable example is the use of AI systems in prostate cancer grading, which has been shown to reduce inter-pathologist variability and improve diagnostic reproducibility [16]. Additionally, recent reviews have highlighted the potential of generative AI chatbots in urology, emphasizing their emerging role in improving patient care and supporting healthcare professionals through natural language models [17].

Our study represents the first evaluation of ChatGPT-4o performance as a decision-support tool in a urological tumor board within a real clinical environment. The overall agreement of 56.1% indicates a moderate-to-good concordance with MTB recommendations (Cohen’s kappa = 0.61); however, its performance varies considerably depending on tumor type, disease stage, clinical context, and treatment modality.

The highest agreement was observed in bladder and renal tumors, as well as in standardized therapeutic scenarios, particularly radiotherapy for localized prostate cancer and biochemical recurrence. In contrast, discrepancies were more frequent in metastatic prostate cancer, a context that requires nuanced clinical judgment integrating tumor burden, age, comorbidities, and treatment sequencing. A recurring source of discrepancy involved misclassification of metastatic volume according to CHAARTED criteria [15], leading to inappropriate therapeutic suggestions.

Analysis by treatment modality revealed that radiotherapy cases had the highest full agreement rate (67.6%), followed by surgical interventions (50%). In contrast, hormonotherapy responses were universally correct but incomplete—indicating a lack of specificity regarding regimen details—while immunotherapy and active surveillance scenarios exhibited lower concordance rates.

Taken together, these findings indicate that ChatGPT-4o demonstrates greater reliability in structured, protocol-driven clinical contexts, whereas its performance declines in complex scenarios requiring integration of multimodal information—such as metastatic disease, treatment sequencing, or nuanced radiologic findings. This pattern aligns with prior evidence showing reduced large language model performance in advanced-stage or guideline-sparse settings [18,19,20,21,22].

Importantly, statistical analysis did not reveal significant differences in agreement based on tumor type, stage, or clinical context; however, descriptive trends suggest that certain subgroups—such as metastatic prostate cancer or diagnostic/follow-up discussions—are more prone to discordance or incomplete recommendations. Interpretation of subgroup findings should be cautious, as certain categories—such as testicular and upper urinary tract tumors—were represented by single cases, precluding meaningful statistical comparison.

These findings also raise important considerations regarding clinical safety. Some of the disagreements identified in our study—particularly in metastatic prostate cancer—could lead to overtreatment, undertreatment, or inappropriate sequencing of systemic therapies if applied without expert oversight. As with other LLMs, ChatGPT-4o may generate confident but inaccurate recommendations (“hallucinations”), especially in complex or poorly defined clinical scenarios [23,24]. For this reason, AI-generated suggestions should never be used in isolation. Their safe integration into clinical workflows requires continuous supervision by trained specialists, standardized validation protocols, and clear mechanisms to detect and correct potentially harmful outputs.

Disagreement with the multidisciplinary tumor board does not necessarily imply that the AI recommendation was clinically inappropriate. In some cases, discrepancies reflected alternative but potentially acceptable therapeutic approaches or differences in emphasis rather than objectively incorrect management. Furthermore, MTB do not always achieve unanimous consensus, and differing expert opinions may arise during discussion. The reference standard in this study corresponded to the final documented institutional decision, which represents consensus rather than absolute unanimity. Therefore, part of the observed discordance may reflect inherent variability in expert clinical judgment.

Our results are comparable to those reported in other oncologic settings. Lukac et al. found total agreement rates of 61.4% in primary breast cancer, decreasing to 48% in advanced cases [25], figures very similar to our localized and metastatic prostate subgroups. Aghamaliyev et al. [10] reported similar trends in gastrointestinal tumors, where performance declined when decisions required integrating multiple therapeutic modalities. Other authors, such as Vela Ulloa et al. [26] and Schmidl et al. [27], also concluded that although ChatGPT can provide reasonable answers, it lacks the clinical judgment necessary to replace a multidisciplinary tumor board.

Prompt design is another relevant factor. Studies such as Ammo et al. in sarcomas achieved 75.2% agreement through the use of prompt engineering techniques that optimize model instructions [9]. In contrast, our study employed a standardized prompt replicating real-world case presentation, which likely limited performance but enhanced realism and clinical applicability. Similarly, Griewing et al. [28] in breast cancer confirmed that prompt design influences response quality even without advanced techniques.

With 98 real clinical cases, this is the second-largest study in the field—following a previously published study including 115 cases [10]—and the first to include multiple urological tumor types, reflecting the diversity of real-world clinical practice. Most previous studies included smaller, single-tumor samples or simulated cases.

Several limitations must be acknowledged. First, ChatGPT’s internal logic and data sources remain opaque, precluding transparent verification of its clinical reasoning (the “black box” issue) [29,30]. Second, the model may suggest therapies unavailable in certain healthcare systems due to regulatory or accessibility differences. Third, this was a single-center study, and therefore local practice patterns, documentation styles, and institutional workflows may have influenced both the MTB decisions and the model’s performance, limiting the generalizability of the results. Multicenter and international studies will be needed to determine the external validity of these findings. Additionally, no advanced prompt engineering techniques were applied, and all cases were entered in Spanish; given that LLMs often perform optimally in English, linguistic factors may have contributed to some discrepancies. Another important consideration is the dynamic nature of LLMs. As model versions are periodically updated, reproducibility over time may be affected. The present evaluation reflects performance at a specific time point and version (ChatGPT-4o). Future studies should assess temporal stability and inter-version variability before considering clinical deployment.

Finally, this study was retrospective and exploratory, and the AI system did not influence real-time decision-making. Agreement metrics provide an initial measure of alignment but do not assess feasibility, workflow integration, efficiency, or impact on multidisciplinary deliberation. Accordingly, this work should be interpreted as a pilot evaluation aimed at generating real-world evidence and identifying practical limitations. We are currently conducting a prospective implementation study to assess real-time integration into tumor board workflows, response stability across repeated queries, and performance in complex clinical scenarios. Such analyses will be essential to define the true clinical utility, safety profile, and operational feasibility of LLMs in multidisciplinary oncologic care.

Uro-oncology tumor boards typically address complex cases requiring collaboration among multiple specialties, a resource often limited to tertiary centers. As a result, access to expert tumor boards remains uneven, particularly in rural or resource-limited settings [31,32]. In this scenario, scalable AI tools such as ChatGPT could help democratize oncologic knowledge, facilitating the dissemination of standardized recommendations and providing support in initial planning, second opinions, and continuous clinical training Although these tools cannot replace multidisciplinary expertise, their responsible and supervised integration has the potential to improve equity, optimize resources, and expand access to high-quality oncologic care, bringing clinical excellence closer to any healthcare setting [12].

## 5. Conclusions

ChatGPT-4o demonstrated an overall agreement rate of 56.1% with tumor board decisions, performing better in standardized therapeutic scenarios and showing greater limitations in complex metastatic cases. However, AI systems are not a substitute for multidisciplinary tumor boards or expert clinical judgment. As these models evolve and become more accurate, they could consolidate their role as supportive tools or automated second opinions, contributing to greater efficiency and consistency in oncologic treatment planning.

## Figures and Tables

**Table 1:** Global agreement scale.

Category	Description
(1) Correct and complete	Absolute agreement both in the number of options provided by the AI and in their quality.
(2) Correct but incomplete	The AI’s response is correct but may: Offer multiple options without focusing on one. Omit a relevant option.
(3) Some correct, others not	The AI provides some correct options but includes others that are incorrect.
(4) Incorrect and incomplete	The response is completely erroneous, with no correct elements in number or content.

## Data Availability

Data supporting the reported results are available from the corresponding author upon reasonable request.

## Supplementary Materials

The following supporting information can be downloaded at: https://www.mdpi.com/article/10.3390/jcm15062130/s1.
