# Supplementary material for: Evaluation of Artificial Intelligence as a Decision-Support Tool in Urological Tumor Boards: A Study in Real Clinical Practice
Source: J Clin Med. 2026 Mar 11;15(6):2130. doi: 10.3390/jcm15062130 (PMC13027246; doi:10.3390/jcm15062130)
Supplement: Supplementary file 1 [file jcm-15-02130-s001.zip › jcm-4167350-supplementary.pdf]

Supplementary Materials

1. Full Prompt Used to Query ChatGPT-4o

Below is the full prompt used consistently for all clinical cases evaluated in the study:

FULL PROMPT

"You are participating as an expert assistant in a multidisciplinary urologic tumor board (MTB). You will receive anonymized clinical information about a patient, including demographic data, medical history, tumor characteristics, staging, and the specific clinical question posed to the MTB.

Your task is to:

1. Provide a management recommendation consistent with current international urologic oncology guidelines (EAU, NCCN, AUA),
2. Justify your decision in a concise and structured manner,
3. List reasonable alternative options, if applicable.

Important instructions:

- Do not request additional information.
- If any information is missing or ambiguous, assume it is *not available* and base your reasoning solely on the data provided.
- Do not make up or infer clinical details beyond what is explicitly stated.
- Offer your final recommendation clearly under the heading: 'Final MTB Recommendation'.

Please analyze the following case:"

2. Example of Case Input Provided to ChatGPT-4o

Patient demographic information:

- Sex: Male
- Age: 72 years
- Relevant comorbidities: Hypertension, type 2 diabetes
- ECOG: 1

Tumor characteristics:

- Diagnosis: Prostate adenocarcinoma
- Gleason score: 4+5

- PSA at presentation: 145 ng/mL
- Imaging: Bone scan shows ≥4 osseous metastases including vertebrae and pelvis

Clinical question:
Based on the information provided and your most up-to-date knowledge on the subject, what is the optimal diagnostic and/or therapeutic approach for this specific clinical scenario?”
Note: If any variable in the original MTB dataset was not available (e.g., exact PSA, date of diagnosis, mutation status), the case was entered as: ‘Information not available’.

3. Handling of Missing or Ambiguous Clinical Information

- Clinical information was transcribed exactly as documented in the MTB case records.
- No assumptions, interpretations, or imputations were made by the investigators.
- When a data point was not available in the original MTB notes, the input explicitly stated: ‘Information not available’.
- Ambiguous descriptions (e.g., 'possible progression', 'clinical suspicion', 'limited metastases') were reproduced verbatim.
- ChatGPT-4o was instructed not to request additional information and to base its recommendation solely on the case description provided.
